# Efficacy and safety of the combination of camrelizumab and apatinib in the treatment of liver cancer: a systematic review and single-arm meta-analysis

**DOI:** 10.1186/s12876-024-03144-8

**Published:** 2024-01-31

**Authors:** Min Chen, Yanglei Li, Minyu Cheng

**Affiliations:** Department of Pharmacy, Zhejiang Hospital, No.12 Ling Yin Road, Hangzhou, 310013 Zhejiang China

**Keywords:** Liver cancer, Camrelizumab, Apatinib, Meta-analysis

## Abstract

**Objective:**

To evaluate the efficacy and safety of the combination of camrelizumab and apatinib in the treatment of liver cancer and to furnish clinical recommendations for pharmacological interventions.

**Methods:**

PubMed, Embase, Web of Science and the Cochrane Library were scrutinized for research publications from their inception to 22 December 2023. Bibliographic perusal and data procurement were executed. The quality of the included studies was evaluated employing the MINORS tool. Meta-analysis was conducted utilizing Stata 15.0 software.

**Results:**

A total of 10 studies involving 849 patients were included in the meta-analysis. The study revealed that the objective response rate (ORR) of the combined therapy was 28% (95% CI: 23%-34%), the disease control rate (DCR) was 69% (95% CI: 64%-73%), the median progression-free survival (mPFS) was 5.87 months (95% CI: 4.96–6.78), the median overall survival (mOS) was 19.35 months (95% CI: 17.53–21.17), the incidence of any grade adverse events was 90% (95% CI: 85%-95%), and the occurrence of grade 3 or higher adverse events was 49% (95% CI: 27%-71%).

**Conclusion:**

The combination of camrelizumab and apatinib exhibits commendable effectiveness in the management of liver cancer; nevertheless, vigilance should be exercised concerning potential adverse reactions in clinical applications to enhance the safety of pharmacological interventions.

## Introduction

Liver cancer constitutes one of the most prevalent malignant neoplasms globally, ranking 6th in incidence amidst all cancers and 3rd in fatalities, exhibiting the most accelerated escalation in mortality throughout the past several decades [1–3]. Hepatocellular carcinoma accounts for the highest proportion of liver cancer cases, ranging from 75 to 85% [4, 5]. The onset of liver cancer is often latent, and the preponderance of patients have already advanced to intermediate or progressive stages at the time of initial detection, thereby losing the prospects for surgical intervention and localized therapy. Systemic pharmacological intervention typically constitutes the sole recourse, and the swift advancement of immune checkpoint inhibitors (ICIs) has introduced novel therapeutic alternatives and engendered optimism for patients afflicted with intermediate and advanced liver cancer [6–8]. Nonetheless, studies [9, 10] have ascertained that the impact of ICI monotherapy on hepatic neoplasms is less than optimal, thereby inciting inquiries into concomitant therapy with molecularly targeted pharmaceuticals. Targeted agents reconfigure the neoplastic immune microenvironment, efficaciously amplifying the potency of immunotherapy and yielding a synergistic outcome [11, 12]. The initial clinical ramifications of the conjunction of the immune checkpoint inhibitor camrelizumab and the antiangiogenic inhibitor apatinib have manifested as auspicious, and this strategy has surfaced as a novel trajectory in the therapeutic landscape of hepatic malignancies. Camrelizumab, a programmed cell death receptor-1 (PD-1) inhibitor, operates by impeding the interplay between PD-1 and its cognate ligand, programmed cell death ligand-1 (PD-L1), subsequently interrupting the immunosuppressive pathway exploited by malignant entities. This revitalizes the immunological response, reestablishes immune surveillance capabilities, and generates sustained anti-neoplastic effects. At present, camrelizumab has exhibited propitious results in the clinical handling of classical Hodgkin's lymphoma, hepatocellular carcinoma, pulmonary neoplasms, and esophageal squamous cell carcinoma [13, 14]. Apatinib, a vascular endothelial growth factor receptor-2 (VEGFR-2) antagonist, functions by impeding the phosphorylation of VEGFR-2, thereby attenuating downstream signalling cascades and curbing tumour angiogenesis to exert its anti-neoplastic properties. This agent has demonstrated promising therapeutic outcomes in advanced gastric adenocarcinoma, gastroesophageal junction adenocarcinoma, and hepatocellular carcinoma [15, 16]. Presently, emerging clinical investigations suggest that the combination therapy of camrelizumab and apatinib may offer certain advantages in the clinical management of liver cancer [15, 16]. However, the precise therapeutic efficacy and safety profile of this regimen remain to be conclusively established [17]. Consequently, this study conducted a comprehensive systematic review and meta-analysis to evaluate the efficacy and safety of camrelizumab in conjunction with apatinib for the treatment of liver cancer, with the aim of providing evidence-based guidance for clinical practice.

## Materials and methodology

### Literature search

The search encompassed databases such as PubMed, Embase, Web of Science, the Cochrane Library, and ClinicalTrials.gov, spanning from their inception to 22 December 2023. Search terms incorporated "Hepatocellular Carcinomas", "Liver Cancer", "Liver Cell Carcinoma", "camrelizumab", "SHR-1210", "apatinib", "rivoceranib" and "YN-968D1", utilizing both MeSH terms and free-text queries.

We have applied for the PROSPERO registration (CRD42023442948).

### Inclusion and exclusion criteria

#### Inclusion criteria

(1) Eligible patients were aged 18 years or older with histopathologically or cytologically confirmed hepatocellular carcinoma or radiologically assessed by enhanced computed tomography or magnetic resonance imaging combined with detection of serum tumour markers; (2) The intervention under investigation is the combined treatment of camrelizumab and apatinib; (3) Studies must report efficacy endpoints and adverse events, encompassing an objective response rate (ORR), disease control rate (DCR), median overall survival (mOS), median progression-free survival (mPFS),adverse events (AEs) and grade 3 or higher adverse events (AEs); (4) Study designs comprise randomised control trials, non-randomised control trials and single-arm studies, etc.

#### Exclusion criteria

(1) Animal and in vitro experiments, basic research; (2) Conference abstracts, reviews, commentaries, case reports; (3) Aggregate reporting of results from multiple populations or disease cohorts; (4) Duplicate publications; (5) Literature from which valid outcome data cannot be extracted.

### Data extraction

Two investigators independently assessed the titles and abstracts of identified publications, performing full-text analysis on eligible articles to determine their final inclusion. Disagreements were resolved through discussions involving a third reviewer. Key information extracted from the original studies encompassed: (1) basic information about the included studies, such as author details, publication dates, and study design; (2) fundamental characteristics of study participants, including total sample size and age and gender distribution of enrolled cases; (3) specific intervention approaches and follow-up durations; (4) pertinent outcome measures; (5) information required for literature quality appraisal.

### Literature quality assessment

Given that the most included studies were single-arm trials, the Methodological Index for Non-Randomized Studies (MINORS) assessment criteria were employed for literature quality evaluation [18]. The assessment entailed 12 indicators, with the first eight (I-VIII) pertaining to single-arm studies without a control group. The numbers I-VIII in the assessment criteria mean: I, a clearly stated objective; II, inclusion of consecutive patients; III, prospective data collection; IV, endpoints appropriate to the objective of the study; V, unbiased assessment of the study endpoint; VI, follow-up period appropriate to the study objective; VII, loss to follow-up less than 5%;VIII, prospective calculation of study size. Each indicator was scored on a scale of 0–2 points: 0 points denoted non-reporting, 1 point signified reported but with insufficient information and 2 points indicated reported with adequate information, and a very objective assessment of each indicator was made. A final score of 13–16 points indicated high-quality studies, and 9–12 points denoted medium-quality studies. According to the MINORS appraisal instrument, this meta-analysis incorporated solely literature of intermediate to high quality.

### Statistical analysis methods

Stata 15.0 software was utilized to perform the statistical analysis of the extracted data. The odds ratio (OR) was used for dichotomous variables and the mean difference (MD) was used as the combined effect statistic for continuous variables. The effect size of all pooled results was reported as a 95% confidence interval (CI) with upper and lower limits. The heterogeneity of the included studies was assessed using *I*^*2*^ and Cochran's Q test. A fixed-effects model was implemented for analysis when *I*^*2*^ ≤ 50% and P ≥ 0.1. In contrast, when *I*^*2*^ > 50% and *P* < 0.1, indicating significant study heterogeneity, a random-effects model was adopted for analysis. The sensitivity analysis was performed for the pooled results with high heterogeneity. In addition, meta-regression is used to further explore the sources of heterogeneity. The collective findings were visually depicted using forest plots. The potential publication bias was scrutinized utilizing Egger's test, with a *P* > 0.05, suggesting an absence of significant publication bias.

## Results

### Retrieval results and fundamental characteristics of the literature

**Fig. 1:**
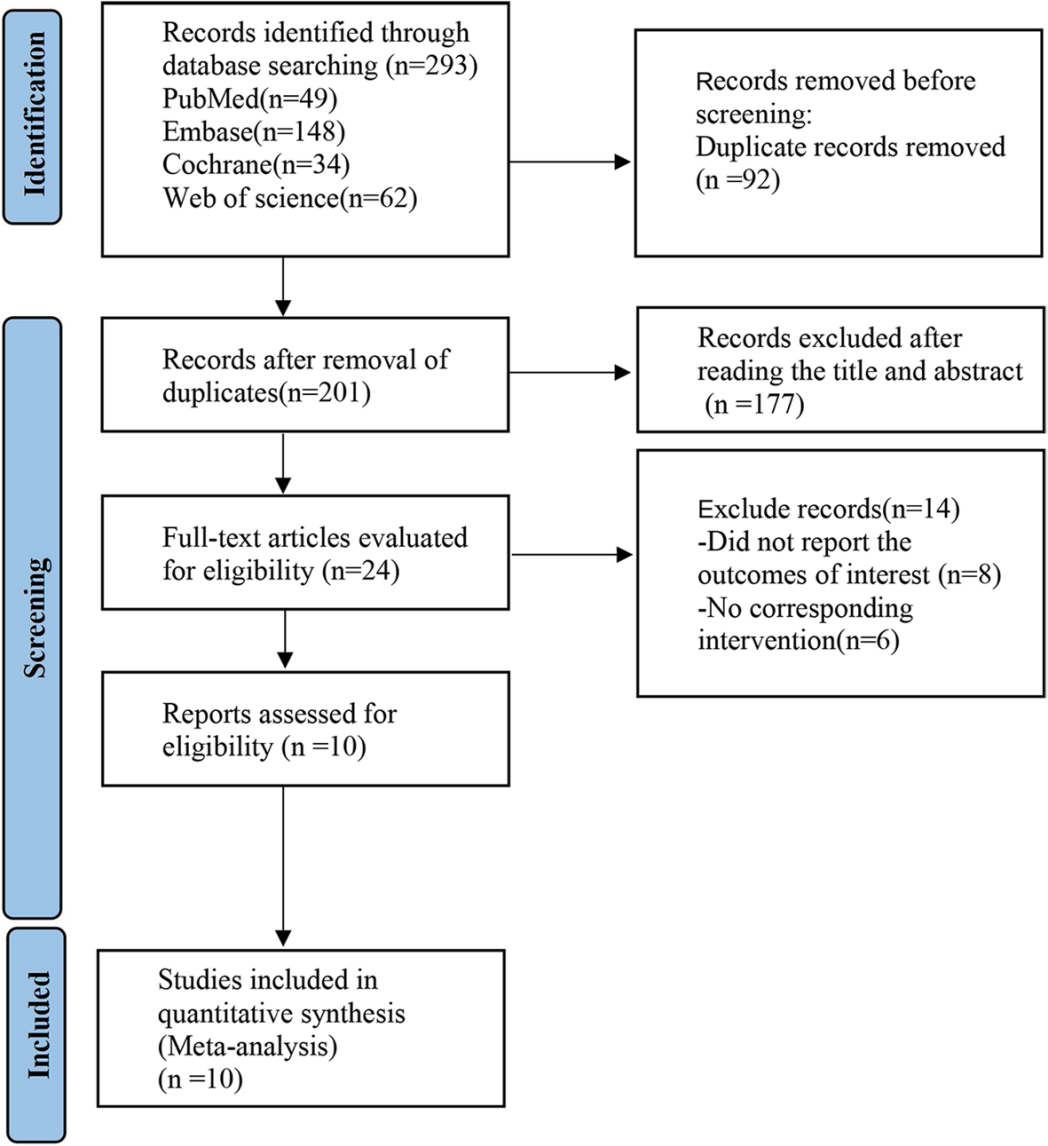
PRISMA flow diagram of the study process. PRISMA, Preferred Reporting Items for Systematic review and Meta-analysis

The search in collective databases yielded a total of 293 pertinent articles. After rigorous screening based on the inclusion and exclusion criteria, 92 duplicates were removed. Furthermore, based on a thorough assessment of their titles and abstracts, an additional 177 irrelevant articles were discarded. Ultimately, ten articles [19–28]were deemed eligible for analysis following a meticulous examination of their full text. The detailed flowchart outlining the literature screening process is demonstrated in Fig. 1, while the essential characteristics of the selected articles are comprehensively depicted in Table 1.

**Table 1:** Depiction of the fundamental characteristics of the selected articles

Study	Year	Country	Diagnosis	Type	Sample size	Gender (M/F)	Mean age (years)	Child–Pugh(A/B)	Intervention	Follow-up Time (months)	Outcome
Shuguang Ju [20]	2021	China	unresectable HCC	RCT	52	44/8	55	41/11	camrelizumab + apatinib	13.5	ORR, DCR, mPFS, mOS, AEs
Guosheng Yuan [25]	2021	China	unresectable HCC	A single-arm study	86	72/14	55	64/22	camrelizumab + apatinib	NR	ORR, DCR, AEs
Jianming Xu [21]	2021	China	advanced HCC	A single-arm study	190	169/21	53	162/28	camrelizumab + apatinib	16.7	ORR, mPFS, AEs
Zhiming Zeng [23]	2021	China	advanced liver cancer	A single-arm study	45	35/10	52	38/7	camrelizumab + apatinib	NR	ORR, DCR, AEs
Yongxiang Xia [24]	2022	China	HCC	A single-arm study	18	17/1	54.7	NR	camrelizumab + apatinib	NR	ORR, AEs
Kuimin Mei [22]	2021	China	advanced primary liver cancer	A single-arm study	21	18/3	48	20/1	camrelizumab + apatinib	12.4	ORR, DCR, mOS, mPFS, AEs
Jianming Xu [19]	2019	China	advanced HCC	A single-arm study	18	17/1	49	13/5	camrelizumab + apatinib	7.9	ORR, DCR mPFS, AEs
Guosheng Yuan [26]	2020	China	HCC	A single-arm study	63	58/5	48	54/9	camrelizumab + apatinib	12.6	ORR, DCR, mPFS, AEs
Shukui Qin [27]	2023	China	unresectable or metastatic HCC	RCT	272	227/45	58	236/36	camrelizumab + apatinib	14.5	ORR, mPFS, mOS, AEs
Dongbo Chen [28]	2023	China	HCC	A single-arm study	86	77/9	57	NR	camrelizumab + apatinib	13.5	ORR, DCR, mPFS, mOS, AEs

### Literature quality assessment

The most included articles were single-arm studies; thus, the MINORS criteria were employed for quality assessment. The results of the quality evaluation are presented in Table 2.

**Table 2:** Quality assessment of the included studies

Study	I	II	III	IV	V	VI	VII	VIII	Total
Shuguang Ju (2021) [20]	2	2	2	2	2	2	2	1	15
Guosheng Yuan (2021) [25]	2	2	1	2	2	1	2	1	13
Jianming Xu (2021) [21]	2	2	2	2	1	2	2	1	14
Zhiming Zeng (2021) [23]	2	2	1	2	1	1	2	2	13
Yongxiang Xia (2022) [24]	2	2	1	2	2	1	1	1	12
Kuimin Mei (2021) [22]	2	2	1	2	1	2	1	2	13
Jianming Xu (2019) [19]	2	2	1	2	1	2	1	2	13
Guosheng Yuan (2020) [26]	2	2	2	2	1	2	1	2	14
Shukui Qin (2023) [27]	2	2	1	2	2	2	2	2	15
Dongbo Chen (2023) [28]	2	2	1	2	2	1	2	2	14

MINORS index for included non-randomized studies

Numbers I-VIII in heading signified:I, a clearly stated aim;II, inclusion of consecutive patients;III, prospective collection of data; IV, endpoints appropriate to the aim of the study; V, unbiased assessment of the study endpoint;VI, follow-up period appropriate to the aim of the study;VII, loss of follow up less than 5%;VIII, prospective calculation of the study size

### Therapeutic efficacy indicators

#### Objective Response Rate (ORR)

**Fig. 2:**
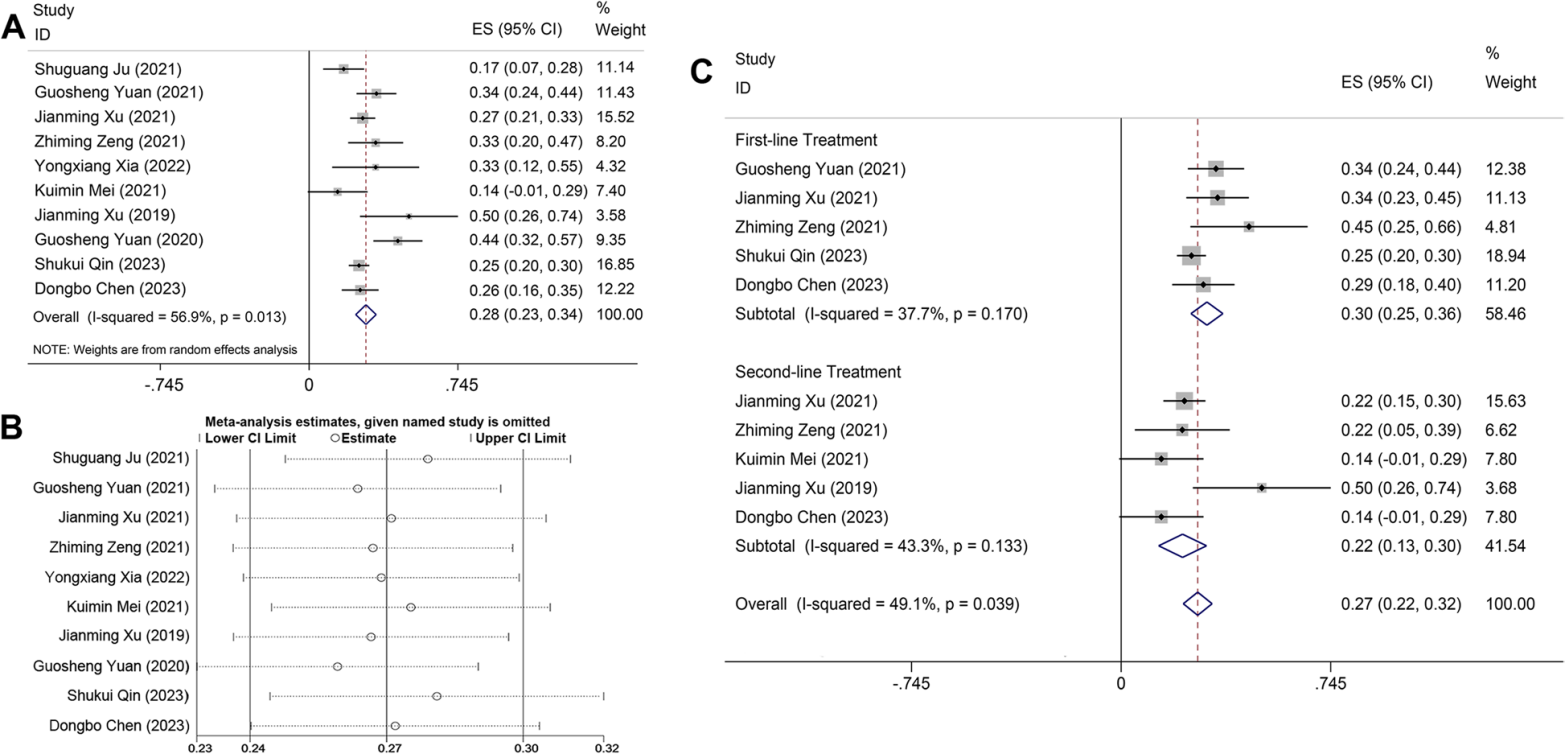
**A** Forest plot delineating ORR of camrelizumab in combination with apatinib for liver cancer treatment; **B** Sensitivity analysis on ORR of camrelizumab in combination with apatinib for liver cancer treatment; **C** Forest plot of subgroup analysis on ORR of camrelizumab in combination with apatinib as first-line or second-line therapy for liver cancer

In total, 10 publications were incorporated, encompassing 849 patients, with 239 individuals attaining objective disease remission. The aggregated analysis indicated that the ORR of camrelizumab combined with apatinib for liver cancer was 28% (95% CI: 23%-34%, *I*^*2*^ = 56.9%, *p* = 0.013), as depicted in Fig. 2A. As *I*^*2*^ = 56.9% > 50%, the random effects model was selected for the analysis, and the sensitivity analysis was continued to test the source of heterogeneity, the results of the sensitivity analysis showed good stability of the study, as shown in Fig. 2B. Moreover, a supplementary subgroup analysis of the objective remission by first-line and second-line therapies was performed. The findings revealed that the ORR for camrelizumab combined with apatinib as a first-line intervention was 30% (95% CI: 25%-36%, *I*^2^ = 37.7%, *p* = 0.170), while for second-line therapy, it was 22% (95% CI: 13%-30%,* I*^2^ = 43.3%, *p* = 0.133), as illustrated in Fig. 2C, suggesting that this combined strategy exhibits a higher objective remission rate when employed as first-line treatment for liver cancer.

#### Disease Control Rate (DCR)

**Fig. 3:**
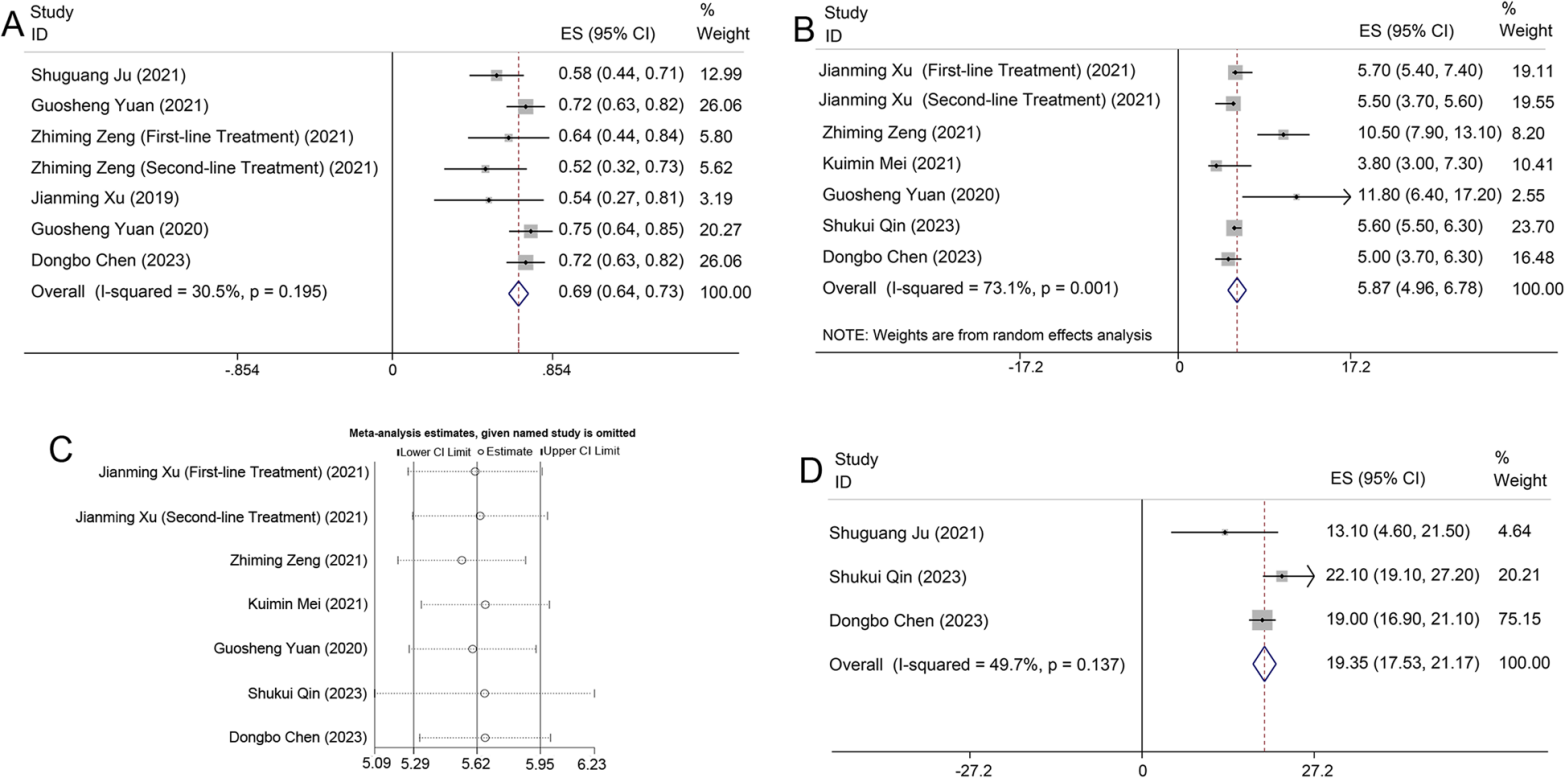
**A** Forest plot illustrating DCR of camrelizumab combined with apatinib for liver cancer treatment; **B** Forest plot delineating mPFS of camrelizumab in combination with apatinib for liver cancer treatment; **C** Sensitivity analysis on mPFS of camrelizumab in combination with apatinib for liver cancer treatment; **D** Forest plot illustrating mOS of camrelizumab combined with apatinib for liver cancer treatment

Altogether, 6 articles comprising 7 research groups were included (Zhiming Zeng (2021) was subdivided into first-line and second-line treatment cohorts), totalling 345 patients, with 234 individuals exhibiting controlled disease progression. The aggregated analysis revealed that the DCR of camrelizumab combined with apatinib for liver cancer treatment was 69% (95% CI: 64%-73%, *I*^2^ = 30.5%, *p* = 0.195), as depicted in Fig. 3A.

#### Median Progression-Free Survival (mPFS)

In total, 6 articles encompassing 7 research groups were incorporated (Jianming Xu (2021) was categorized into first-line and second-line treatment cohorts), and the aggregated analysis indicated that the mPFS of camrelizumab combined with apatinib for liver cancer treatment was 5.87 months (95% CI: 4.96–6.78, *I*^2^ = 73.1%, *p* = 0.001), as illustrated in Fig. 3B. As *I*^2^ = 73.1%, we selected the random effects model for the analysis and continued the sensitivity analysis to test the source of heterogeneity. The results of the sensitivity analysis showed good stability of the analysis, as shown in Fig. 3C.

#### Median Overall Survival (mOS)

Three studies were incorporated into the statistical analysis, and the results demonstrated that the mOS of camrelizumab combined with apatinib for liver cancer treatment was 19.35 months (95% CI: 17.53–21.17, *I*^2^ = 49.7%, *p* = 0.137), as depicted in Fig. 3D.

### Safety

A comprehensive analysis of the adverse reaction incidence rates for liver cancer treatment utilizing camrelizumab combined with apatinib was conducted, encompassing 6 articles and a total of 674 patients. Among them, 622 patients encountered general adverse reactions, with an occurrence rate of 90% (95% CI: 85%-95%, *I*^2^ = 92.8%, *p* = 0.000); 455 patients experienced grade 3 or higher adverse reactions, with an incidence rate of 49% (95% CI: 27%-71%, *I*^2^ = 97.7%, *p* = 0.000), as delineated in Table 3. Due to the high level of heterogeneity, we performed meta-regression analyses by study design, which showed any grade AEs (*p* = 0.939) and grade 3 or higher AEs (*p* = 0.229), as delineated in Table 4, indicating that study design covariate was not significantly associated with PFS and OS and other factors may be at play. Predominantly, the general adverse reactions with a higher incidence encompass Thrombocytopenia (51%, 95% CI: 41%-62%, *I*^2^ = 71.3%, *p* = 0.031), Hypertension (45%, 95% CI: 27%-62%,*I*^2^ = 95.6%, *p* = 0.000), and Hand-foot skin reaction (45%, 95% CI: 33%-57%, *I*^2^ = 84.1%, *p* = 0.000), in addition to Leukopenia (40%), Proteinuria (37%), Abdominal pain (34%), Diarrhea (31%), Hepatotoxicity (24%), Fever (20%), Hypothyroidism (20%), RCCEP (19%), Rash (18%), Fatigue (17%), and Nausea and vomiting (11%), as delineated in Table 3. Primarily, severe adverse reactions with a higher incidence include Hypertension (19%, 95% CI: 4%-34%, *I*^2^ = 98.5%, *p* = 0.000), Thrombocytopenia (9%, 95% CI: 1%-17%, *I*^2^ = 88.7%, *p* = 0.000), and Hand-foot skin reaction (6%, 95% CI: 3%-9%, *I*^2^ = 32.4%, *p* = 0.218), along with Proteinuria (5%), Hepatotoxicity (3%), Abdominal pain (2%), Diarrhea (2%), and Rash (1%), as portrayed in Table 3.

**Table 3:** Meta-analysis outcomes of adverse reactions for liver cancer treatment with camrelizumab combined with apatinib

adverse event	Any grade						Grade ≥ 3					
	study	Heterogeneity		ES (95%CI)	P	P(Egger)	study	Heterogeneity		ES (95%CI)	P	P(Egger)
		P	I^2^(%)					P	I^2^(%)			
Total	6	0.000	92.8	0.90(0.85,0.95)	0.000	0.420	6	0.000	97.7	0.49(0.27,0.71)	0.000	0.250
Thrombocytopenia	3	0.031	71.3	0.51(0.41,0.62)	0.000	0.020	3	0.000	88.7	0.09(0.01,0.17)	0.034	0.277
Hypertension	7	0.000	95.6	0.45(0.27,0.62)	0.000	0.829	5	0.000	98.5	0.19(0.04,0.34)	0.011	0.063
Hand-foot skin reaction	5	0.000	84.1	0.45(0.33,0.57)	0.000	0.201	4	0.218	32.4	0.06(0.03,0.09)	0.000	0.693
Leukopenia	3	0.001	85.8	0.40(0.26,0.55)	0.000	0.422	NR					
Abdominal pain	4	0.017	70.7	0.34(0.24,0.44)	0.000	0.421	3	0.466	0.00	0.02(0.00,0.03)	0.017	0.323
Proteinuria	6	0.000	97.1	0.37(0.17,0.56)	0.000	0.797	4	0.114	49.7	0.05(0.02,0.07)	0.000	0.906
Diarrhea	3	0.711	0.0	0.31(0.26,0.36)	0.000	0.203	2	0.661	0.0	0.02(0.00,0.04)	0.015	-
Hepatotoxicity	2	0.000	97.8	0.24(0.18,0.66)	0.260	-	2	0.589	0.0	0.03(0.01,0.06)	0.003	-
Fever	2	0.003	88.7	0.20(0.15,0.54)	0.261	-	NR					
Hypothyroidism	6	0.000	85.2	0.20(0.12,0.27)	0.000	0.616	NR					
Rash	5	0.000	87.8	0.18(0.09,0.27)	0.000	0.653	4	0.195	36.2	0.01(0.00,0.03)	0.057	0.088
Fatigue	4	0.163	41.4	0.17(0.11,0.22)	0.000	0.843	NR					
RCCEP	4	0.003	78.3	0.19(0.10,0.27)	0.000	0.295	NR					
Nausea and vomiting	3	0.885	0.0	0.11(0.08,0.14)	0.000	0.453	NR

**Table 4:** Results of meta-regression analyses for adverse events in liver cancer treatment with camrelizumab in combination with apatinib

Outcome	Study	Factor	Heterogeneity		P	SE
			P	I^2^(%)		
AEs (any grade)	6	Study Type	0.000	92.8	0.939	-0.0281683 to 0.0304537
AEs(grade ≥ 3)	6	Study Type	0.000	97.7	0.229	-0.2439073 to 1.020582

AEs (any grade), any treatment-related adverse event (grade 1–2); AEs(grade ≥ 3), any treatment-related adverse event (grade 3–5)

### Publication bias analysis

An analysis of publication bias was executed on the incorporated studies utilizing the Egger test. The results revealed that ORR (*p* = 0.268), DCR (*p* = 0.068), mPFS (*p* = 0.469), mOS (*p* = 0.828), incidence of general adverse reactions (*p* = 0.420), and incidence of ≥ 3-grade adverse reactions (*p* = 0.250) conformed to the criterion of *p* > 0.05. This implies that no significant publication bias exists within the study.

All of the above results are summarised in Table 5.

**Table 5:** Summary of meta-analysis results

Outcome	Study	Heterogeneity		ES (95%CI)	P	P(Egger)
		P	I^2^(%)			
ORR (%)	10	0.013	56.9	0.28(0.23,0.34)	0.000	0.268
DCR (%)	7	0.195	30.5	0.69(0.64,0.73)	0.000	0.068
mPFS(months)	7	0.001	73.1	5.87(4.96,6.78)	0.000	0.469
mOS(months)	3	0.137	49.7	19.35(17.53,21.17)	0.000	0.828
AEs (any grade, %)	6	0.000	92.8	0.90(0.85,0.95)	0.000	0.420
AEs (grade ≥ 3, %)	6	0.000	97.7	0.49(0.27,0.71)	0.000	0.250

*ORR* the objective response rate, *DCR* the disease control rate, *mPFS* the median progression-free survival, *mOS* the median overall survival, *AEs* (any grade) any treatment-related adverse event (grade 1–2), *AEs* (grade ≥ 3) any treatment-related adverse event (grade 3–5)

## Discussion

In recent years, the burgeoning development of immunosuppressive agents has ushered liver cancer treatment into a new epoch of immunotherapy [29]. Particularly, the collaborative strategy with antivascular targeted therapy has demonstrated promising application potential in liver cancer clinical treatment, offering new therapeutic hopes for patients afflicted with liver cancer [30–33]. Research indicates that the synergy between immunotherapy and anti-vascular targeted therapy yields an augmented antitumor effect [34, 35]. Anti-angiogenic drugs can facilitate the infiltration and activation of immune cells within tumours, mediate the upregulation of IFNγ, enhance the expression of PD-1 and PD-L1, boost the sensitivity of immunotherapy within tumours, alter the M1/M2 ratio of tumour-associated macrophages, diminish the infiltration of regulatory T cells and monocytes in tissues, restructure the tumour immune microenvironment, and effectively elevate the efficacy of immunotherapy [11, 12]. Furthermore, immunosuppressive agents may trigger the recruitment of immune subpopulations possessing vascular regulatory activity, potentially serving as a target for anti-angiogenic treatment [36]. Consequently, the combined utilization of both can ameliorate the local vascular microenvironment, effectively eradicate tumour cells, and jointly enhance clinical treatment outcomes. Among these, the PD-1 inhibitor camrelizumab combined with the VEGFR-2 inhibitor apatinib has exhibited favourable treatment prospects in the clinical management of liver cancer.

Through a comprehensive analysis of the ten incorporated articles in this study, it was discovered that the clinical efficacy of camrelizumab combined with apatinib in treating liver cancer is commendable. This combination not only yields satisfactory objective remission rates and disease control rates but also provides patients with significant benefits in terms of median progression-free survival and median overall survival. Moreover, through an in-depth subgroup analysis, it was discerned that the objective remission rate of the combined regimen as first-line therapy was considerably higher compared to second-line therapy. On the one hand, this could be attributed to the diminished functional status of patients when their second-line treatment was adopted, which results in a lower tolerable drug dosage, thereby directly reducing the therapeutic effect of the combined regimen. On the other hand, prior treatment may render the immune microenvironment within the patient’s body increasingly complex. For instance, if first-line treatment has already involved immunosuppressive agents, it might lead to the development of drug-resistant antibodies, engendering resistance to immunotherapy [37] and subsequently weakening the therapeutic effect of second-line medications. Nonetheless, due to the limited number of studies presently incorporated in the analysis, the research findings necessitate more clinical trial data and a larger sample size for validation and substantiation.

The results of CARES-310, a global phase 3 randomized open-label trial on camrelizumab plus apatinib indicate that this combination therapy presents a promising first-line treatment option for unresectable HCC with a positive benefit-to-risk profile. This study is the first to report significant benefits in progression-free survival and overall survival with the combination of an anti-PD-1 antibody and an oral small molecule anti-angiogenic agent as first-line treatment for unresectable HCC, compared to sorafenib. However, it is important to acknowledge the limitations of this study, including its open-label design and the fact that the majority of participants were from Asia and had hepatocellular carcinoma of viral aetiology. Further research is required to confirm the effectiveness of the treatment in other patient subgroups [27]. Although camrelizumab in combination with apatinib in the treatment of HCC has achieved a high probability of being the most effective treatment in terms of both OS and PFS, more direct comparative research analysis with existing standard first-line treatments is needed in the future [38, 39]. Furthermore, in the analysis of adverse reactions, it was ascertained that the overall safety profile of the combined regimen is generally acceptable. However, it may also provoke the risk of adverse reactions in patients, such as thrombocytopenia, hypertension, and hand-foot skin reaction. Meanwhile, immune-related adverse events such as liver damage also deserve clinical attention. Immune-related liver injury during treatment with ICI is relatively common in patients with HCC and is often detected by elevated ALT/AST levels [40]. Therefore, it is crucial to closely monitor relevant clinical symptoms and indicators through a more comprehensive clinical examination, such as cardiovascular function tests, routine blood tests and liver function tests, etc., so that the physician can adjust the dosage of the drug and undertake appropriate interventions to ensure clinical efficacy and safety. In addition, multidisciplinary management, which requires integrated collaboration between the specialties involved in the management of patients with HCC, is valuable. For example, treatment for HCC patients is administered after multidisciplinary assessment and according to the practice of participating institution, and continued until disease progression or unacceptable toxicity. Toxicity management, including dose modification, is performed in accordance with the summary of product characteristics for the agents [40]. The development of multidisciplinary care for HCC patients is essential to optimise the management of treatment side effects and improve patient outcomes.While this study endeavoured to incorporate as many pertinent studies as possible that fulfil the criteria, it still exhibits the following limitations: 1. Variations in disease subtypes, medication dosage, sample size, follow-up duration, and statistical methods contribute to increased research heterogeneity; 2. It is unable to acquire comprehensive data for additional subgroup analyses; 3. Some included studies have a brief follow-up period, not attaining overall survival (OS) and progression-free survival (PFS); 4. The most included studies are single-arm trials with smaller sample sizes, necessitating larger-scale, multicentre, randomized controlled clinical trials for combined analysis and evaluation, with the aim of providing more objective and efficacious evidence-based medicine for clinical treatment.

## Conclusion

In conclusion, camrelizumab in combination with apatinib shows a favourable therapeutic effect and a manageable safety profile in HCC. Further investigation can delve into the effective biomarkers of this combined regimen, identify the optimal treatment population, and thus achieve precision and individualization in liver cancer therapy. Simultaneously, it may be worthwhile to explore combining this treatment with surgical procedures, radiofrequency ablation, and interventional therapies to afford liver cancer patients a broader array of treatment options and opportunities, thereby harnessing its clinical potential and value.

## Data Availability

All data generated or analyzed during this study are included in this published article.
